# Abnormal functional connectivity in the right dorsal anterior insula associated with cognitive dysfunction in patients with type 2 diabetes mellitus

**DOI:** 10.1002/brb3.2553

**Published:** 2022-05-11

**Authors:** Man Wang, Dongsheng Zhang, Jie Gao, Fei Qi, Yu Su, Yumeng Lei, Zhirong Shao, Kai Ai, Min Tang, Xiaoling Zhang

**Affiliations:** ^1^ Department of MRI Shaanxi Provincial People's Hospital Xi'an People's Republic of China; ^2^ Xi'an Medical University Xi'an Shaanxi People's Republic of China; ^3^ Philips Healthcare Xi'an Shaanxi People's Republic of China

**Keywords:** cognitive dysfunction, dorsal anterior insula, resting‐state functional magnetic resonance imaging, type 2 diabetes mellitus

## Abstract

**Introduction:**

Type 2 diabetes mellitus (T2DM) is a chronic disease with a high incidence worldwide. T2DM can cause cognitive impairment, but its neuropathological basis is unclear. A variety of neuropsychiatric studies have found that abnormal functional connectivity (FC) in the central executive network (CEN), default‐mode network (DMN), and salience network (SN) may be the neuropathological basis of cognitive dysfunction. The right dorsal anterior insula (dAI) is the core SN area. It plays an important role in regulating the CEN and the DMN. However, few studies have explored the relationship between cognitive impairment and FC among the right dAI, CEN, and DMN in patients with T2DM.

**Methods:**

Resting‐state functional magnetic resonance imaging was used to investigate FC between the right dAI and the CEN and DMN in 44 patients with T2DM and 41 sex‐, age‐, and education‐matched healthy controls, as well as its relationship with clinical/cognitive variables.

**Results:**

In patients with T2DM, FC between the right dAI and multiple brain regions of the CEN and DMN was generally decreased, and FC strength between the right dAI and the inferior frontal gyrus negatively correlated with trail making test A score (*r* = −0.421, *p *= 0.004).

**Conclusions:**

Patients with T2DM exhibit abnormal FC between the right dAI and the CEN and DMN. This may be one of the neuromechanisms of cognitive impairment in patients with T2DM. In addition, reduced FC between the right dAI and the right inferior frontal gyrus may be related to abnormal attention regulation in patients with T2DM.

## Graphical abstract

In the current study, we used resting‐state fMRI to explore FC between the right dAI and the CEN and DMN in patients with T2DM. The results show that FC between the right dAI and several brain regions of the CEN and DMN was decreased in patients with T2DM, suggesting that disconnection of the right dAI is related to the neuropathological mechanism of cognitive impairment in patients with T2DM.

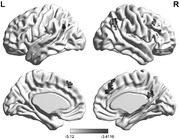

## 1 INTRODUCTION

Type 2 diabetes mellitus (T2DM) is a common chronic metabolic disease in middle‐aged and older people. It is characterized by long‐term hyperglycemia and insulin resistance. At present, the number of affected patients worldwide is as high as 451 million, and the incidence is increasing rapidly (Cho et al., 2018). Patients with T2DM often present with cognitive impairment, such as impairment in episodic memory, attention, and executive function(D. Zhang, Qi, et al., 2020; Qi et al., 2017; X. Zhang et al., 2021), which may increase the risk of Alzheimer's disease (AD)(Crane et al., 2013). Studies have shown that cognitive impairment in patients with T2DM is related to abnormal functional connectivity (FC) in different brain regions (D. Zhang, Qi, et al., 2020; L. Liu, Li et al., 2017; Sun et al., 2018; Tan et al., 2019). However, the neuropathological mechanisms of cognitive dysfunction in T2DM are still not clear.

Neuroscientists consider that cognitive function relies on interactions between brain regions in multiple neural networks. The central executive network (CEN), default‐mode network (DMN), and salience network (SN) play an important role in maintaining normal cognitive function (Menon, 2011). Aberrant organization and interconnectivity of the CEN, SN, and DMN are prominent features of many mental and neurological diseases, such as AD (Li et al., 2019) and mild cognitive impairment(Chand et al., 2017).Triple network model of psychopathology proposes that deficits in engagement and disengagement in the three core neural cognitive networks play a significant role in many mental and neurological diseases, and emphasizes the core position of SN in three cores neural cognitive networks, for initiating network switching, leading to the engagement of CEN and the disengagement of DMN (Menon, 2011).

SN can identify the most salient stimuli among external inputs and internal events, so as to reasonably allocate cognitive resources, coordinating the activation status of the CEN and DMN (Menon & Uddin, 2010; Seeley et al., 2007; Sridharan et al., 2008). In salience processing, the SN is right dominant. Research by Sridaran and colleagues, as well as subsequent work, showed that the right dorsal anterior insula (dAI) plays a key causal role in switching/engaging or disengaging between the two major networks (the CEN and the DMN) and is a key node for initiating network switching/activation (Sridharan et al., 2008; Supekar & Menon, 2012; Uddin et al., 2011). Affected to the right dAI can lead to abnormal saliency processing, resulting in disorderly FC between the SN, CEN, and DMN, affecting cognitive function and behavioral activities (Li et al., 2019; Moran et al., 2013; Uddin et al., 2015).

Previous neuroimaging studies have confirmed a reduction in insula gray matter volume and abnormal spontaneous neuronal activity in patients with T2DM (J. Liu, Liu et al., 2017; Xia et al., 2017). These studies indicate that the insula is one of the most vulnerable brain regions in patients with T2DM. In addition, Liu et al. reported that FC between the right insula and the superior parietal lobule and precentral gyrus/postcentral gyrus of the CEN is decreased (L. Liu, Li et al., 2017). Several studies have demonstrated abnormal FC between the insula and the DMN brain regions (posterior cingulate and medial prefrontal cortex) in patients with T2DM (D. Zhang, Gao, et al., 2020; Tan et al., 2019). These studies demonstrated altered FC between the insula and the CEN and DMN regions. Yang et al. used seed points to explore FC within and between five neural networks, including the SN, CEN, and DMN, in patients with T2DM. They revealed extensively affected FC between these three networks, and the right insula was the most severely affected node in patients with T2DM and mild cognitive impairment (Yang et al., 2016). However, the region of interest (ROI) chosen in these studies was not the right dAI.

Considering the unique role of the right dAI in salience processing, it is necessary to explore FC between the right dAI and the CEN and DMN to understand the neuropathological mechanism of cognitive impairment in patients with T2DM. Seed‐based FC analysis could reveal FC in specific brain regions according to prior anatomical knowledge or activation maps (Lowe et al., 2002), so it can reveal patterns of FC (aberrant or not) with the rest of the brain. Therefore, this study aimed to use the resting‐state functional connectivity (rs‐FC) method to observe FC between the right dAI and the CEN and DMN in patients with T2DM. We hypothesized that the right dAI in patients with T2DM has abnormal FC with the CEN and DMN, and speculate that there is a correlation between abnormal FC, cognitive scores, and clinical variables.

## 2 MATERIALS AND METHODS

### 2.1 Study subjects

A total of 48 patients with T2DM and 46 sex‐, age‐, and education‐matched healthy controls (HCs) were enrolled from June 2019 to November 2020. All the subjects were Han Chinese. Patients were recruited from the Department of Endocrinology, Shaanxi Provincial People's Hospital, while HCs were recruited via advertisements during the same period. Detailed medical history and routine examinations were performed for all subjects. Demographic and clinical data were collected for all subjects, including laboratory tests, blood pressure, body mass index (BMI), education level, alcohol consumption, smoking status, and disease duration (for patients with T2DM only). Blood samples were obtained by venipuncture at 8:00 a.m. after overnight fasting for at least 8 h, to measure the levels of fasting blood glucose, glycated hemoglobin (HbA1c), and blood lipids.

The inclusion criteria were as follows: (1) >40 years of age, right‐handed, and at least 6 years of education; (2) diagnosis of T2DM according to the 2014 criteria of the American Diabetes Association; (3) a fasting blood glucose level of <6.1 mmol/L (HCs only). Exclusion criteria for all subjects included (1) a history of acute metabolic complications, severe hyperglycemia episodes (blood glucose >33.3 mmol/L), or severe hypoglycemia episodes (blood glucose <3.9 mmol/L); (2) cerebrovascular accident, tumor, trauma, infection, or congenital abnormal brain development on conventional brain magnetic resonance imaging (MRI), including white matter score greater than grade 2; (3) major medical conditions, such as anemia, cancer, and thyroid dysfunction; (4) psychiatric or neurologic illness that could influence cognitive function, such as severe depression and Parkinson's disease; (5) a history of stroke or alcohol or other substance dependence; (6) mini‐mental state examination (MMSE) score of <24 (HCs only); (7) contraindications to MRI.

The study was performed in accordance with the Declaration of Helsinki, was approved by the ethics committee of Shaanxi Provincial People's Hospital. All subjects were informed of the test content and methods in detail and provided written informed consent before MRI. All subjects were scanned at 6:00 p.m. on the same day that clinical data were collected and neuropsychological tests were performed. The total scan time of per participant is about 50 min.

### 2.2 Neuropsychological tests

Before functional MRI (fMRI), all subjects completed a detailed standardized cognitive assessment covering multiple cognitive domains. The MMSE and the Montreal cognitive assessment (MoCA) were used to evaluate general cognitive function. Neural response speed and attention were measured using the trail making test‐A (TMT‐A). In the TMT‐A test, participants are asked to correctly link 25 randomly placed encircled numbers on a page. This test was used as internal control tasks to evaluate selective attention and distracted‐attention (distraction). Visuospatial ability was assessed using the clock‐drawing test (CDT). All tests were conducted in 40 min by systemically trained psychiatrists.

### 2.3 MRI image acquisition

All MRI data were acquired using a 3.0‐T MR scanner (Ingenia, Philips Healthcare, the Netherlands) with a 16‐channel phased‐array head coil. Subjects were instructed to lie quietly, keep their eyes closed without falling asleep, and avoid thinking of anything during scanning. Soft cushions were placed on both sides of the coil to fix the head and minimize artefacts caused by head movement. Earplugs were given to reduce scanner noise. Conventional brain axial T1‐weighted, T2‐weighted, and fluid‐attenuated inversion recovery imaging were acquired to exclude visible brain lesions. Images were obtained using resting‐state fMRI and a gradient‐echo planar sequence with the following parameters: repetition time (TR) = 2000 ms, echo time (TE) = 30 ms, number of slices = 34, slice thickness = 4 mm, gap = 0 mm, field of view (FOV) = 230 × 230 mm, acquisition matrix = 128 × 128, flip angle (FA) = 90°, and 200 volumes. Sagittal three‐dimensional T1‐weighted images were acquired with the following parameters: TR = 7.5 ms, TE = 3.5 ms, FA = 8°, FOV = 250 × 250 mm, acquisition matrix = 256 × 256, slice thickness = 1 mm, no gap, and 328 sagittal slices.

### 2.4 Image preprocessing

Preprocessing of fMRI data was performed using Data Processing Assistant for Resting‐State fMRI software, which is based on Statistical Parametric Mapping (SPM12, http://www.fil.ion.ucl.ac.uk/spm) and the toolbox for Data Processing & Analysis of Brain Imaging (http://rfmri.org/DPABI). The first 10 volumes of fMRI images were removed to avoid heterogeneity in the initial MRI signal. The remaining volumes were preprocessed according to the following steps. First, slice timing adjustment and realignment were performed to correct differential slice acquisition times and head motion, respectively. Any subjects with a head motion of >1.5 mm of translation or a 1.5° rotation in any direction were excluded. Then, fMRI data were spatially normalized to the standard Montreal Neurological Institute (MNI) space and resampled into 3 × 3 × 3 mm^3^ voxels, smoothing by a Gaussian filter of 6‐mm full‐width at half‐maximum. Linear detrending and band‐pass filtering (0.01−0.08 Hz) were applied to remove the effects of low‐frequency physiological drift and high‐frequency noise. In the end, nuisance covariates, including 24 head motion parameters, cerebrospinal fluid signal, white matter signal, and global mean signal, were regressed by multiple linear regression analysis.

Finally, four patients with T2DM and five HCs with obvious head motion were excluded from this study. A total of 44 subjects (31 males and 13 females) were included in the T2DM group, while 41 subjects (27 males and 14 females) were included in the control group.

### 2.5 Region of interest definition and FC analysis

According to prior literature (Deen et al., 2011), ROI with a radius of 6 mm were defined as centered in the right dAI (MNI = 35, 7, 3) in the MNI152 space. The mean time course of the ROI was calculated. Then, Pearson's correlation coefficients between the mean time series of the ROI and the time series of other voxels in the whole brain for each subject were calculated. Correlation coefficients were converted to *Z*‐values using the Fisher r‐to‐z transformation to improve normality. *Z*‐values represent the strength of FC between the voxel and the ROI.

In the analysis between groups, the CEN and DMN in GIFT software (http://icatb. sourceforge.net/, version 2.0d) were superimposed to generate a template as a mask for analysis between groups. Two independent samples *t*‐test was performed on the rs‐FC results of the two groups based on DAPBI software to identify brain regions with significant differences in functional connectivity with ROI. The voxel statistical threshold was set at *p *< .001 and a minimum cluster size of 22 voxels, which corresponded to a corrected *p* value of < .05 (Gaussian random field correction [GRF]).

### 2.6 Statistical analysis

SPSS 18.0 was used to conduct the statistical analysis. Two‐tailed independent samples *t*‐tests were used for normally distributed variables. Nonnormally distributed data were evaluated using the Mann–Whitney *U* test. The chi‐squared (*χ* ^2^) test was used to assess intergroup differences in sex. The significance level was set at *p* < .05.

The mean FC value of functionally altered brain regions between the two groups was extracted. The Pearson's correlation coefficients between all the different regions and all cognitive scores and clinical variables were analyzed by SPSS 18.0. The significance level was set at *p* < .05. The correlations were also corrected for age, sex, and education. Then Bonferroni corrections were used for multiple comparisons.

## 3 RESULTS

### 3.1 Clinical data and neuropsychological results

Clinical data and neuropsychological results of patients with T2DM and HCs are presented in Table 1. There were no significant differences in age, sex, education level, BMI, systolic blood pressure, diastolic blood pressure, triglyceride concentration, total cholesterol concentration, high‐density lipoprotein cholesterol concentration, low‐density lipoprotein cholesterol concentration, or MMSE, and CDT scores between the two groups (*p *> .05). Moreover, patients with T2DM had significantly higher HbA1c and fasting blood glucose levels compared with HCs (*p* < .001), while MoCA scores in patients with T2DM were significantly lower compared with HCs (*p* < .05), TMT‐A scores in patients with T2DM were significantly higher compared with HCs (*p* < .05).

**TABLE 1:** Demographic, clinical, and neuropsychological test data of patients with type 2 diabetes mellitus and healthy controls

	T2DM (*n *= 44) (mean ± SD)	HCs (*n *= 41) (mean ± SD)	*p* value
Age (years)	55.18 ± 6.25	54.41 ± 4.96	0.534
Sex (M/F)	31/13	27/14	0.649^#^
Education (years)	13.95 ± 2.57	14.85 ± 2.40	0.100
BMI (kg/m^2^)	24.47 ± 2.92	24.19 ± 2.96	0.659
T2DM duration (years)	9.41 ± 5.28	–	–
FBG (mmol/L)	8.67 ± 2.72	5.25 ± 0.78	<0.000*
HbA1c (%)	8.09 ± 1.80	5.58 ± 0.54	<0.000*
Systolic BP (mmHg)	129.34 ± 21.16	122.43 ± 7.99	0.053
Diastolic BP (mmHg)	81.70 ± 14.00	81.71 ± 5.55	0.999
TG (mmol/L)	1.88 ± 1.25	1.81 ± 1.23	0.803
TC (mmol/L)	4.41 ± 1.15	4.84 ± 0.83	0.061
HDL‐C(mmol/L)	1.10 ± 0.20	1.23 ± 0.38	0.054
LDL‐C (mmol/L)	2.52 ± 0.71	2.81 ± 0.88	0.094
MMSE	28.57 ± 1.35	28.46 ± 1.55	0.713
MoCA	25.47 ± 2.79	26.82 ± 1.64	0.009*
TMT‐A (seconds)	77.47 ± 32.72	70.38 ± 29.23	0.039*
CDT	19.05 ± 8.49	20.97 ± 6.46	0.269

*Note*: Values distributed normally or nonnormally are presented as mean ± SD or median (minimum, maximum).

Abbreviations: BMI, body mass index; BP, blood pressure; CDT, Clock‐Drawing Test; F, female; FBG, fasting blood glucose; HbA1c, glycated hemoglobin; HC, healthy control; LDL‐C, low‐density lipoprotein cholesterol; M, male; MMSE, Mini‐Mental State Examination; MoCA, Montreal Cognitive Assessment; TC, total cholesterol; TG, triglyceride; TMT‐A, Trail Making Test‐A; T2DM, type 2 diabetes mellitus.

^#^ Pearson's *χ* ^2^ test.

* *p* < .05.

### 3.2 Intergroup differences in FC

**FIGURE 1:**
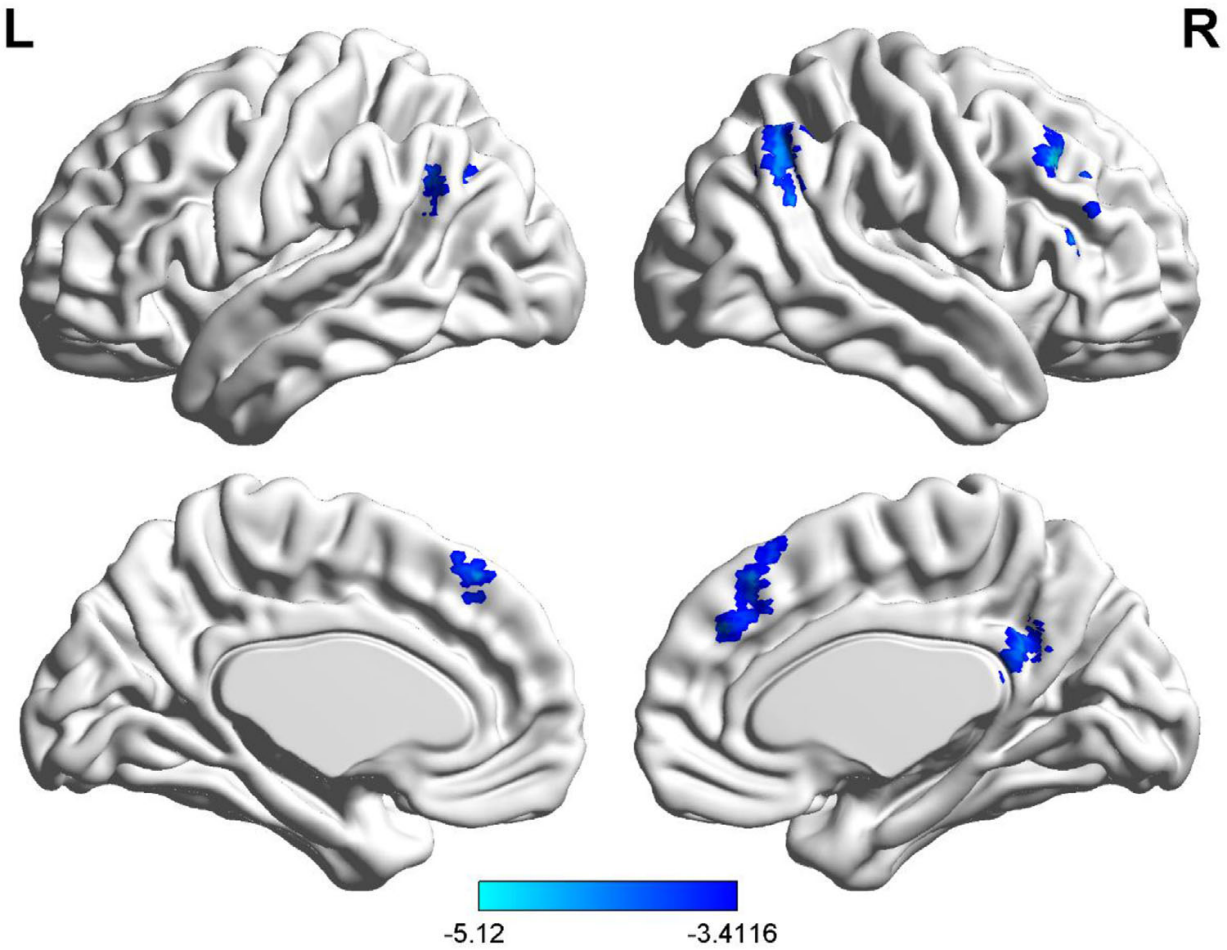
Brain regions with decreased functional connectivity in patients with T2DM, corrected by GRF (threshold of *p* < .001, corrected threshold of *p* < .05). GRF, Gaussian random field correction; T2DM, type 2 diabetes mellitus

Compared with HCs, FC between the right dAI and the right inferior frontal gyrus, right middle frontal gyrus, and right precuneus/posterior cingulate, bilateral medial prefrontal gyrus, bilateral angular gyrus was decreased in patients with T2DM (GRF correction, *p *< .05; Table 2 and Figure 1).

**TABLE 2:** Aberrant functional connectivity in patients with type 2 diabetes compared with healthy controls

		MNI coordinates					
Brain region	BA	X	Y	Z	*T* value	Cluster size	*p*
R inferior frontal gyrus	46	54	33	15	−4.409	34	<0.05
R middle frontal gyrus	9	39	24	39	−5.122	55	<0.05
R precuneus/posterior cingulate cortex	31	3	−51	27	−4.325	57	<0.05
B medial prefrontal cortex	8	−3	36	48	−4.443	82	<0.05
L angular gyrus	39	−42	−63	36	−4.453	45	<0.05
R angular gyrus	40	48	−60	42	−4.372	82	<0.05

Abbreviations: B, bilateral; BA, Brodmann's area; L, left; MNI, Montreal Neurological Institute; R, right.

### 3.3 Correlation between FC and clinical/cognitive variables

**FIGURE 2:**
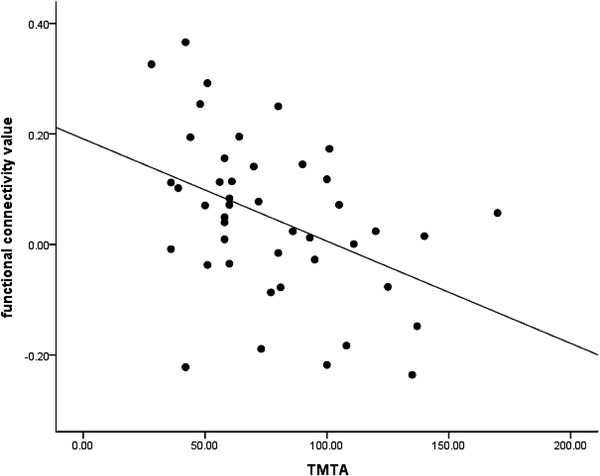
Correlation analysis of FC strength between the right dAI and the right inferior frontal gyrus with TMT‐A score in patients with T2DM (*r* = −0.421, *p* = .004). dAI, dorsal anterior insula; FC, functional connectivity; T2DM, type 2 diabetes mellitus; Trail Making Test‐A

In patients with T2DM, FC strength between the right dAI and the right inferior frontal gyrus was negatively correlated with TMT‐A score (*r* = −0.421, *p *= .004) after Bonferroni correction only (Figure 2), no significant correlation was found in other cognitive scores and clinical variables. After having been corrected for age, sex, and education, FC strength between the right dAI and the right inferior frontal gyrus was still negatively correlated with TMT‐A score (*r* = −0.536, *p *= .000).

## 4 DISCUSSION

In the current study, we used resting‐state fMRI to explore FC between the right dAI and the CEN and DMN in patients with T2DM. The results show that FC between the right dAI and several brain regions of the CEN and DMN was decreased in patients with T2DM. In addition, reduced FC between the right dAI and right inferior frontal gyrus may be related to the patient's attentional deficits.

### 4.1 dAI reduced from CEN regions

The typical pathological basis of cognitive dysfunction in AD is the deposition of β‐amyloid and tau proteins (Lemche, 2018). The pathological mechanism of cognitive impairment in T2DM is believed to be similar to that of AD (Bedse et al., 2015). Studies have found that the hub regions in AD patients are more likely to deposit β‐amyloid (Crossley et al., 2014; Scheinin et al., 2009; Zhou et al., 2012), and insulin resistance can accelerate this process (Verdile et al., 2015). The anterior insula and the DLPFC (BA9/46) are the core brain regions of the SN and CEN, respectively (Habas et al., 2009), this may be the reason for the abnormal FC between anterior insula and DLPFC in T2DM patients.

The anterior insula and DLPFC constitute a cognitive control network (Cole & Schneider, 2007), which is the neural basis for individuals to effectively identify targets, manipulate and process related information, and guide behavior (Badre, 2011). Decreased FC between the right dAI and the DLPFC suggests that internal FC in the cognitive control network in patients with T2DM is disordered. Studies have found that impairment in cognitive control may lead to a series of cognitive processing abnormalities, such as abnormalities in attention distribution, working memory, and reactive inhibition (Lesh et al., 2011). The MoCA can assess general cognitive function in a variety of cognitive units. A study has found that the decreased FC between right DLPFC and insula was negatively correlated with the patient's MOCA score (Hu et al., 2020). In this study, the MoCA score of patients with T2DM was significantly lower compared with HCs. Therefore, we speculated that dysfunction in the cognitive control network in patients with T2DM might be one of the reasons for cognitive decline in these patients.

The cognitive control network plays an important role in target‐oriented behavior(Wu et al., 2020) and is involved in initiation and control of attention (Cole & Schneider, 2007; Seeley et al., 2007). The dAI is a key brain region for switching between internal and external attention (Sridharan et al., 2008) and the inferior frontal gyrus participates in the top‐down attentional control process (Voegler et al., 2016). In the process of attention, the anterior insula is related to detection of salient stimuli and initiation of attention control signals, and the DLPFC is responsible for maintaining these processes (D'Esposito & Postle, 2015; Myers et al., 2017). Decreased FC between the right anterior insula and the inferior frontal gyrus may result in an individual's inability to effectively adjust external attention information (Voegler et al., 2016).

In our study, we found that FC strength between the right dAI and the inferior frontal gyrus negatively correlated with TMT‐A score in patients with T2DM. TMT‐A is often used to evaluate attentional selection (Baschi et al., 2019). This suggests that decreased FC between the right dAI and the inferior frontal gyrus may be related to abnormal attention adjustment in patients with T2DM.

### 4.2 dAI reduced from DMN regions

Episodic memory is mainly maintained by the DMN (McCormick et al., 2014), in which the posterior cingulate, precuneus, medial prefrontal cortex, and angular gyrus constitute a core network responsible for episodic memory retrieval (recall). The network plays a key role in successfully initiating retrieval and integrating recall content into a cohesive memory representation (Kim, 2010; King et al., 2015; Rugg & Vilberg, 2013). Research indicates that the insula is also involved in the retrieval phase of episodic memory (Daselaar et al., 2001; Kim, 2010; Spaniol et al., 2009). In addition, FC between the right posterior cingulate and the insula is positively correlated with episodic memory performance, both in immediate and delayed recall (Viard et al., 2019). In this study, we found that right dAI had reduced FC with multiple brain regions that make up the episodic memory retrieval network, which may indicate abnormal episodic memory retrieval in patients with T2DM. We compared MMSE and MoCA scores for immediate and delayed memory units between groups, and found that the delayed memory scores of T2DM patients were lower than those of HC group (See Table S2), which confirmed our speculation to a certain extent. Although previous studies have confirmed that abnormalities in episodic memory in patients with T2DM (Sadanand et al., 2016). Our study may provide new insight to explore the neuroimaging mechanisms of episodic memory impairment in patients with T2DM.

### 4.3 Limitations

This study has the following limitations. First, most patients with T2DM in this study were revisited patients with a longer disease course, so the results of this study may not be applicable to patients with a shorter disease course. Second, some patients with T2DM have different complications and different medical conditions (See Tables S1 and S3), which may influence the results. Future research should distinguish these patients to avoid this possibility bias. Third, the cognitive scale in this study is not comprehensive enough. Although the cognitive scores of memory units in MMSE and MoCA are different, the efficacy is low, thus, we will improve the scale in future studies to further confirm our conclusions. Although there was no gender difference between groups in this study, male subjects were significantly more than female subjects in both T2DM and HC groups. Sala et al (2019) showed that gender may have some influence on brain metabolism and neural connectivity. Therefore, we will increase the sample size and balance the gender ratio of each group to in future studies to make the research results more rigorous.

## 5 CONCLUSION

In conclusion, decreased FC between the right inferior frontal gyrus in the CEN may underpin functional impairment in attention. Decreased FC between the right dAI and the DMN in multiple brain regions may help us to understand the underlying neuromechanisms of episodic memory impairment in patients with T2DM from another perspective.

## CONFLICT OF INTEREST

The authors declare no conflict of interest.

### PEER REVIEW

The peer review history for this article is available at https://publons.com/publon/10.1002/brb3.2553

## Supporting information

SUPPORTING INFORMATION

SUPPORTING INFORMATION

## Data Availability

The data that support the findings of this study are available on re‐quests from the corresponding author.
