# Supplementary material for: Abnormal functional connectivity in the right dorsal anterior insula associated with cognitive dysfunction in patients with type 2 diabetes mellitus
Source: Brain Behav. 2022 May 11;12(6):e2553. doi: 10.1002/brb3.2553 (PMC9226846; doi:10.1002/brb3.2553)
Supplement: Supplementary file 1 — SUPPORTING INFORMATION [file BRB3-12-e2553-s001.docx]

***Supplementary Material***

**Supplementary Table 3.** T2DM complications

| Complications | T2DM group |
| --- | --- |
| Retinopathy | 10 |
| Neuropathy | 12 |
| Nephropathy | 12 |
| Cardiovascular disease | 6 |
| Peripheral artery disease | 29 |
| Periodontitis | **-** |
