# Supplementary material for: Abnormal functional connectivity in the right dorsal anterior insula associated with cognitive dysfunction in patients with type 2 diabetes mellitus
Source: Brain Behav. 2022 May 11;12(6):e2553. doi: 10.1002/brb3.2553 (PMC9226846; doi:10.1002/brb3.2553)
Supplement: Supplementary file 2 — SUPPORTING INFORMATION [file BRB3-12-e2553-s002.docx]

***Supplementary Material***

**Supplementary Table 1.** T2DM medical conditions

| Medical conditions | Medication | Number of patients |
| --- | --- | --- |
| diet control |  | 7 |
| Insulin |  | 3 |
|  | Metformin | 13 |
| Oral medication | Metformin + sulfonylureas | 6 |
|  | Metformin + acarbose | 4 |
|  | Sulfonylureas | 6 |
|  | Metformin | 2 |
| Insulin + oral medication | Acarbose | 2 |
|  | Metformin + sulfonylureas | 1 |

T2DM: type 2 diabetes mellitus.

**Supplementary Table 2.** MOCA/MMSE scores for immediate memory and delayed memory

unit in the T2DM and HCs

|  | **T2DM (*n* = 44*)* (mean ± SD)** | **HCs (*n* = 41) (mean ± SD)** | ***p* value** |
| --- | --- | --- | --- |
| MMSE |  |  |  |
| Immediate memory | 3.03± 0.00 | 2.90 ± 0.43 | 0.142 |
| Delayed memory | 2.75±0.53 | 2.95±0.31 | 0.039* |
| MOCA |  |  |  |
| Delayed memory | 3.40±1.45 | 3.97±1.10 | 0.047* |

* *p* < .05. T2DM: type 2 diabetes mellitus, HCs: healthy controls
